# Prominent Heart Organ-Level Performance Deficits in a Genetic Model of Targeted Severe and Progressive SERCA2 Deficiency

**DOI:** 10.1371/journal.pone.0079609

**Published:** 2013-11-04

**Authors:** Frazer I. Heinis, Kristin B. Andersson, Geir Christensen, Joseph M. Metzger

**Affiliations:** 1 Department of Integrative Biology and Physiology, University of Minnesota Medical School, Minneapolis, Minnesota, United States of America; 2 Department of Biochemistry, Molecular Biology, and Biophysics, University of Minnesota, Minneapolis, Minnesota, United States of America; 3 Institute for Experimental Medical Research, Oslo University Hospital Ullevaal and University of Oslo, Oslo, Norway; 4 Center for Heart Failure Research, University of Oslo, Oslo, Norway; University of Otago, New Zealand

## Abstract

The cardiac SERCA2 Ca^2+^ pump is critical for maintaining normal Ca^2+^ handling in the heart. Reduced SERCA2a content and blunted Ca^2+^ reuptake are frequently observed in failing hearts and evidence implicates poor cardiac Ca^2+^ handling in the progression of heart failure. To gain insight into mechanism we investigated a novel genetic mouse model of inducible severe and progressive SERCA2 deficiency (inducible *Serca2* knockout, SERCA2 KO). These mice eventually die from overt heart failure 7-10 weeks after knockout but as yet there have been no reports on intrinsic mechanical performance at the isolated whole heart organ level. Thus we studied whole-organ *ex vivo* function of hearts isolated from SERCA2 KO mice at one and four weeks post-knockout in adult animals. We found that isolated KO heart function was only modestly impaired one week post-knockout, when SERCA2a protein was 32% of normal. At four weeks post-knockout, function was severely impaired with near non-detectable levels of SERCA2. During perfusion with 10 mM caffeine, LV developed pressures were similar between 4-week KO and control hearts, and end-diastolic pressures were lower in KO. When hearts were subjected to ischemia-reperfusion injury, recovery was not different between control and KO hearts at either one or four weeks post-knockout. Our findings indicate that *ex vivo* function of isolated SERCA2 KO hearts is severely impaired long before symptoms appear *in vivo*, suggesting that physiologically relevant heart function *in vivo* can be sustained for weeks in the absence of robust SR Ca^2+^ flux.

## Introduction

Normal heart pump function requires the highly regulated, cyclical release and reuptake of Ca^2+^ in the myoplasm of cardiac myocytes. Reuptake of Ca^2+^ into the sarcoplasmic reticulum (SR), accomplished by the cardiac SR Ca^2+^ ATPase (SERCA2), plays a major role in cardiac muscle relaxation and also is critical in determining SR Ca^2+^ load and subsequent systolic Ca^2+^ release. Defects in Ca^2+^ handling are clearly associated with cardiac dysfunction and heart failure[1]–[6]. Although diminished Ca^2+^ flux and reduced SERCA2a expression are frequently observed in the failing heart, the exact relationship between these observations is not fully known. It is not clearly established whether loss of SERCA2a is a driving primary cause of severe cardiac dysfunction or whether this is a secondary consequence of the emerging cardiac pathology.

*Serca2* ^+/−^ mice, which express approximately 60% of normal SERCA2a protein content, have only mildly impaired function[7],[8],[9]. Given that *Serca2* heterozygous mice represent only one state of diminished SERCA2a activity, a more refined approach to study the relationship between SERCA2 expression and heart function in adult mice is necessary. A recently developed model of inducible SERCA2 knockout, the SERCA2 KO mouse, allows for selective disruption of the *Serca2* gene in the hearts of adult mice[10], [11]. In this model, exons 2 and 3 of *Serca2* gene locus are flanked by loxP sites, thereby allowing selective disruption of *Serca2* in adult cardiac myocytes conferred by a tamoxifen-sensitive, cardiac myocyte-expressed MerCreMer transgene[12].

This model of genetic SERCA2 manipulation has been characterized *in vitro* in isolated cardiac myocyte studies, as well as *in vivo* by survival, echocardiography and invasive micromanometry studies[11], [13], [14]. Following *Serca2* gene disruption, SERCA2a protein is rapidly lost from the heart (t_1/2_ ∼3 days). Despite the emergence of severe cardiac SERCA2a deficiency within weeks, the SERCA2 KO mice survive for up to 10 weeks post-knockout before finally succumbing to end-stage heart failure and pulmonary congestion[13]. Myocytes isolated from SERCA2 KO hearts at 4 and 7 weeks post-knockout reveal severe impairments in contractility, intracellular Ca^2+^ transient magnitude, and Ca^2+^ transient decay rates[11], [13]. The *in vivo* phenotype at these time-points as assessed by echocardiography, however, is surprisingly mild. This suggests that SERCA2 KO mice are capable of compensating, at least temporarily, for the loss of SERCA2 activity for much longer than predicted based on current knowledge of cardiac Ca^2+^ handling and SR function (reviewed in [15]).

The disconnect between severely impaired *in vitro* function of isolated myocytes with the apparent preserved *in vivo* function after *Serca2* disruption is surprising and warrants more detailed study. Interestingly, a recent parallel study using isolated rabbit hearts and the potent SERCA2a inhibitor thapsigargin showed that heart pump function can be sustained, at least in the short term, in absence of any appreciable myocyte SR Ca^2+^ handling capabilities[16]. These findings raise important new questions regarding the precise relationship between SERCA2a deficiency and the progression of heart failure.

Although the contractile function and electrophysiology of isolated myocytes from inducible *Serca2* KO have been studied, and *in vivo* function has been assessed by echocardiography and invasive micromanometry, no studies thus far have reported the contractile function of whole hearts isolated from these mice. It therefore remains unclear whether the whole-organ function of KO hearts will most closely resemble the severely impaired isolated myocyte phenotype or the largely preserved *in vivo* hemodynamic phenotype. In order to address this gap in knowledge, we isolated hearts at specific time-points after cardiac *Serca2* gene disruption and directly examined whole heart performance. We hypothesized that, similar to isolated myocytes, isolated *Serca2* KO hearts would exhibit severe contractile dysfunction *ex vivo* at times when *in vivo* pathology is not yet manifest. We studied *Serca2* KO and FL control hearts at 1 and 4 weeks post-knockout, time points well prior to the onset of overt pump failure, but where severe dysfunction of isolated cardiacmyocytes is manifest, to establish an organ-level biological SERCA2 dose-response relationship. Since sarcolemmal Ca^2+^ currents are increased following Serca2 knockout[11], [13], [14], we perfused isolated 4-week Serca2 FL and KO hearts with caffeine to reveal SR-independent contractile function. In addition, we used this model to test whether altered SR Ca^2+^ handling could improve the functional recovery of isolated hearts from ischemia-reperfusion injury. Because there are reports that increasing SR Ca^2+^ load can severely impair recovery from ischemic injury *ex vivo* [17], we hypothesized that decreasing SR Ca^2+^ content by *Serca2* disruption would improve the recovery of KO hearts relative to controls.

## Methods

### Animal Handling and Ethics Statement

**Figure 1:**
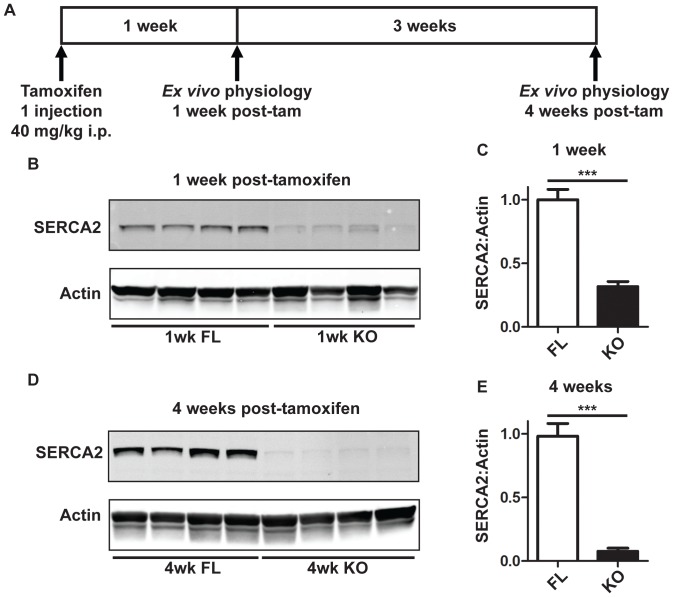
Experimental timeline and SERCA2 protein determination in FL and KO hearts. **A**, Experimental Timeline. All animals were injected with tamoxifen then sacrificed either 1 or 4 weeks later for heart isolation. KO mice expressed the αMHC-MerCreMer transgene, which efficiently excised LoxP-flanked exons of the *Serca2* locus in response to tamoxifen. FL mice received tamoxifen, but retained normal SERCA2 levels due to lack of MerCreMer. **B-C**, at 1 week post-tamoxifen, SERCA2 content in KO hearts was diminished to 32% of FL. **D-E**, at 4 weeks post-tamoxifen, faint SERCA2 bands were observed in 20 µg of heart lysate. ***: P<0.001 for FL vs. KO at either 1 week or 4 weeks post-tamoxifen injection, as determined by unpaired two-tailed *t* test.

All experiments were approved by the University of Minnesota Institutional Animal Care and Use Committee (NIH Animal Welfare Assurance #A3456-01). Mice were housed on a 12-12 hour light-dark cycle and provided rodent chow and tap water *ad libitum*. All mice were homozygous for loxP sites in introns 1 and 3 of the *Serca2* gene (*Serca2^fl/fl^*), and either contained (TG) or did not contain (NTG) the αMHC-MerCreMer^+/o^ transgene. Mice were genotyped as described [11]. All mice were injected with tamoxifen dissolved at 10 mg/ml in peanut oil (1×40 mg/kg intraperitoneally). *Serca2^fl/fl^*; TG:αMHC-MerCreMer^+/o^ mice efficiently deleted *Serca2* in response to tamoxifen and became *Serca2^0^* (KO) in the heart, while *Serca2^fl/fl^*; NTG animals retained the floxed *Serca2* gene and normal SERCA2 expression (FL controls). FL and KO groups were sacrificed by sodium pentobarbital injection (100 mg/kg i.p.) and exsanguination at 1 and 4 weeks post-tamoxifen injection and their hearts were removed for *ex vivo* functional analysis (Figure 1A). All mice were 8–12 weeks of age at the time of sacrifice, and were of both sexes in roughly equal proportion. FL and KO animals were sacrificed 7 days post-tamoxifen for the 1-week time point, and 29–30 days post-tamoxifen for the 4-week time point.

### Langendorff protocol

Mice were anesthetized with sodium pentobarbital (100 mg/kg i.p.) and heparinized (250 IU i.p.). Upon loss of toe pinch reflex, the ribcage was opened and the heart was removed to a dish of ice-cold Krebs-Henseleit solution (KHB: In mmol/L, 118 NaCl, 4.7 KCl, 1.2 MgSO_4_, 1.2 KH_2_PO_4_, 0.5 NaEDTA, 2.5 CaCl_2_, 15 Glucose, 25 NaHCO_3_, 0.5 NaPyruvate). The aorta was trimmed and cannulated, and the heart was mounted on a Langendorff apparatus (Radnoti, Inc) and retrogradely perfused with KHB bubbled with 95% O_2_ / 5% CO_2_ and maintained near 37 °C with a water jacket. The atria were removed, and a balloon catheter was inserted into the left ventricle (LV) to measure isovolumic LV pressure. An electrode placed at the base of the heart controlled pacing frequency, which was set at 7 Hz (pacing cycle length of 1000/7 = 143 milliseconds) for baseline and equilibration.

**Figure 2:**
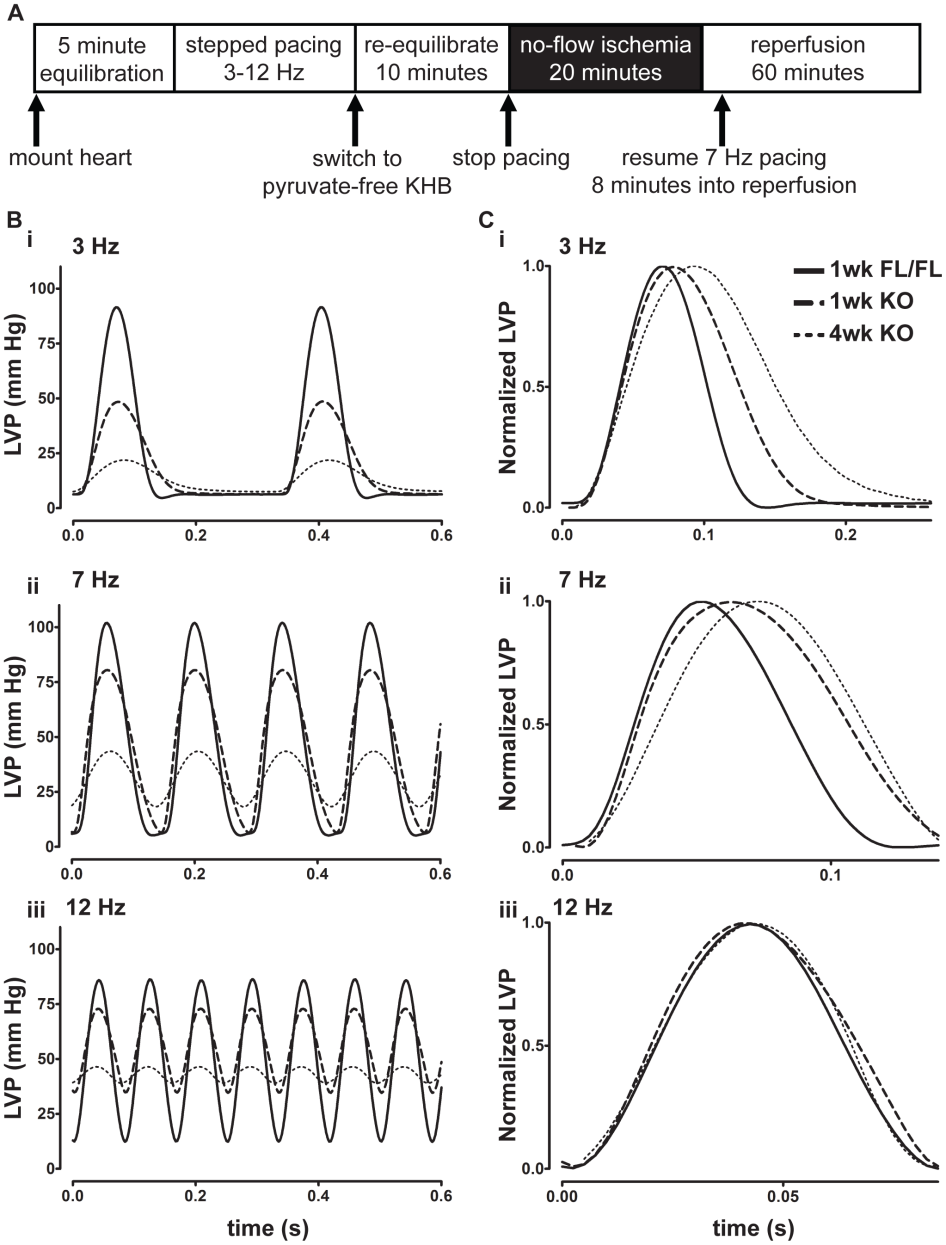
Langendorff protocol and individual LV pressure traces. **A**, Langendorff Protocol. After initial equilibration, pacing frequency was stepped between 3 and 12 Hz in 1-Hz intervals. After pacing at 12 Hz, hearts were re-equilibrated for 10 minutes in KHB lacking pyruvate and then subjected to 20 minutes no-flow ischemia and 60 minutes reperfusion. **B**, Representative traces of LV pressure from 1-week FL (solid line), 1-week KO (dashed line), and 4-week KO (dotted line) hearts sampled over 0.6 seconds. **C**, Individual peaks normalized to developed pressure. Note the differences in time scale for each normalized peak. **i**, **ii**, and **iii**: 3, 7, and 12 Hz traces, respectively, for either absolute LV pressure (**B**) or LV pressure normalized to own developed pressure (**C**).

The Langendorff protocol is presented in Figure 2A. Hearts were equilibrated at 7 Hz for 5 minutes, followed by stepped changes in pacing frequency from 3 to 12 Hz: frequency was first reduced stepwise from 7 Hz to 3 Hz, then returned to 7 Hz and increased stepwise to 12 Hz in 1-Hz increments. Each frequency step was maintained until an equilibrium developed, at which point the frequency was again increased (usually ∼1 minute per step). Parameters from 5 to 10 beats were averaged at this equilibrium state of each pacing step. After pacing steps to 12 Hz were completed, frequency was set at 7 Hz, perfusion was switched to KHB lacking pyruvate, and hearts were re-equilibrated at 7 Hz for 10 minutes to wash out pyruvate from the pacing protocol.

After re-equilibration, pacing was ceased and hearts were subjected to 20 minutes of no-flow ischemia, followed by 60 minutes of reperfusion. 7 Hz pacing was resumed after 8 minutes of reperfusion and continued until the end of the experiment.

### Caffeine perfusion

*Serca2* knockout was performed and 4-week FL and KO hearts were mounted in the Langendorff mode as described above. KHB composition was as above, except glucose was 10 mM and pyruvate was not used. Hearts were allowed to equilibrate in normal KHB for 10 minutes. After equilibration, 5 minutes of baseline performance were recorded and the perfusate was switched to a reservoir containing KHB +10 mM caffeine. Hearts were perfused with KHB + caffeine for 10 minutes, followed by 20 minutes washout in normal KHB. Pacing frequency was 7 Hz throughout. Arrhythmic behavior during and after caffeine perfusion was defined as the time from start of caffeine perfusion until the first 15-second interval in which contractile frequency deviated outside 7±0.5 Hz (420±30 BPM).

### Tissue handling

After the Langendorff protocol was complete, hearts were removed from the cannula, quickly blotted and weighed, and frozen using liquid nitrogen. Frozen hearts were pulverized in a liquid nitrogen-cooled steel deadblow, resuspended in 750 µl RIPA (in mmol/L, 50 Tris, 150 NaCl, 1 EDTA, plus 0.5% w/v SDS and protease inhibitors [1 mM PMSF and 0.001 mg/ml each of aprotinin, leupeptin, and pepstatin, all added fresh]), briefly sonicated, and centrifuged at 14,000x*g* for 2 minutes. Supernatant protein concentration was determined using bicinchroninic acid.

### Western Blotting

Heart lysates were diluted to 1 µg/µl with RIPA and Laemmli buffer, and then 20 µg protein per lane was fractionated on 12.5% Tris-Glycine gels (BioRad Criterion) and transferred to PVDF membranes. SERCA2a and Actin were detected by immunoblot (primary antibodies: SERCA2a, 2A7-A1 (Sigma) at 1∶1000; Actin, A-2103 (Sigma) at 1∶10,000) using an Odyssey imaging system (LiCor, Inc).

### Data Acquisition and Statistical Analysis

Data was acquired using LabChart 6 software (AD Instruments) and analyzed using Prism 5.02 (GraphPad). Significance was tested by unpaired two-tailed *t* test or two-way analysis of variance with Bonferroni post-test, where appropriate. Significance was set at P < 0.05. Data is presented as mean ± SEM unless SEM was smaller than figure icon.

## Results

*Serca2* FL and *Serca2* KO hearts were studied at one and four weeks after tamoxifen injection. Following tamoxifen injection, SERCA2 protein was rapidly lost from the heart (Figure 1). One week post-tamoxifen, SERCA2 protein in KO hearts was 32% of FL control (Figure 1B,C). By four weeks post-tamoxifen, only faint SERCA2 bands were found in heavily loaded samples (Figure 1D). Despite weeks of severe SERCA2a depletion in these mice, there were no overt, readily observable differences in animal appearance and no differences in heart weight, body weight, or heart weight-body weight ratio between FL and KO groups at either the one or four week time points (Table 1).

**Table 1:** Animal Characteristics.

Group	1wk FL	1wk KO	4wk FL	4wk KO
**Heart Weight (mg)**	117±8.6	107±11.0	115±4.3	104±5.2
**Body Weight (g)**	22.6±2.3	21.6±2.2	22.±1.2	20.7±2.2
**HW:BW Ratio (mg/g)**	5.2±0.2	5.0±0.3	5.4±0.2	5.1±0.3
**Age (wks)**	8.7±0.4	8.9±0.5	11.6±0.2	12.0±0.3
**Time Post-Tamoxifen (days)**	7±0	7±0.3	29±0.6	30±0.9

No significant differences in heart weight, body weight, HW:BW ratio, or age were found between FL and KO hearts at 1 and 4 weeks post-tamoxifen injection. Unpaired two-tailed *t* tests were used to compare FL to KO hearts at each time point. Values are mean ± SEM.

Isolated heart contractile parameters at baseline are detailed in Table 2. One week post-tamoxifen, KO hearts exhibited mild systolic and diastolic dysfunction. The minimal first derivative of LV pressure (dP/dt_min_, the fastest rate of pressure decay each beat) was significantly reduced in KO hearts compared to FL. In addition, times to 50% pressure rise (T50% Rise) and fall (T50% Relaxation or T50R) were significantly increased in KO hearts relative to FL at 1 week post-tamoxifen. In comparison, at four weeks post-tamoxifen, KO hearts had severe systolic and diastolic dysfunction relative to FL. In KO hearts LV developed pressure and maximal and minimal dP/dt were markedly decreased, and end-diastolic pressure (LVEDP) and times to 50% rise and 50% relaxation were significantly increased (P<0.05). Representative traces of LV pressure (Figure 2B, LV pressures; 2C, normalized to magnitude) from one-week FL, one-week KO, and four-week KO reveal severe, progressive decline in isolated heart contractile performance as SERCA2 protein is lost from the myocardium. At low to moderate pacing frequencies (3 and 7 Hz, Figure 2Bi-ii and 2Ci-ii), KO hearts contracted more weakly and relaxed more slowly than FL hearts. At higher pacing frequencies, these differences in relaxation performance were diminished. At four-weeks KO heart diastolic function was severely impaired at all pacing frequencies (Figure 2B).

**Table 2:** Baseline Isolated Heart Function.

Group	1wk FL	1wk KO	4wk FL	4wk KO
# Hearts	4	4		5	4
**Developed Pressure (mm Hg)**	99.2±8.5	72.6±7.8 (n.s.)		90.4±5.4	20.4±4.9 (***)
**End-Diastolic Pressure (mm Hg)**	5.9±2.1	7.5±0.8 (n.s.)		5.8±0.4	18.8±2.6 (***)
**dP/dt Max (mm Hg s^−1^)**	3034±268	2232±307 (n.s.)		2512±189	487±120 (***)
**dP/dt Min (mm Hg s^−1^)**	−2441±292	−1359±148 (*)		−2231±190	−401±88 (***)
**T50% Rise (ms)**	25.52±0.63	30.51±1.29 (*)		27.46±0.54	33.95±1.09 (***)
**T50% Relaxation (ms)**	31.16±1.21	41.19±1.26 (**)		30.42±1.09	41.24±0.78 (***)
**FDHM (ms)**	56.68±0.87	71.69±2.42 (**)		57.88±0.94	75.19±1.55 (***)

**Figure 3:**
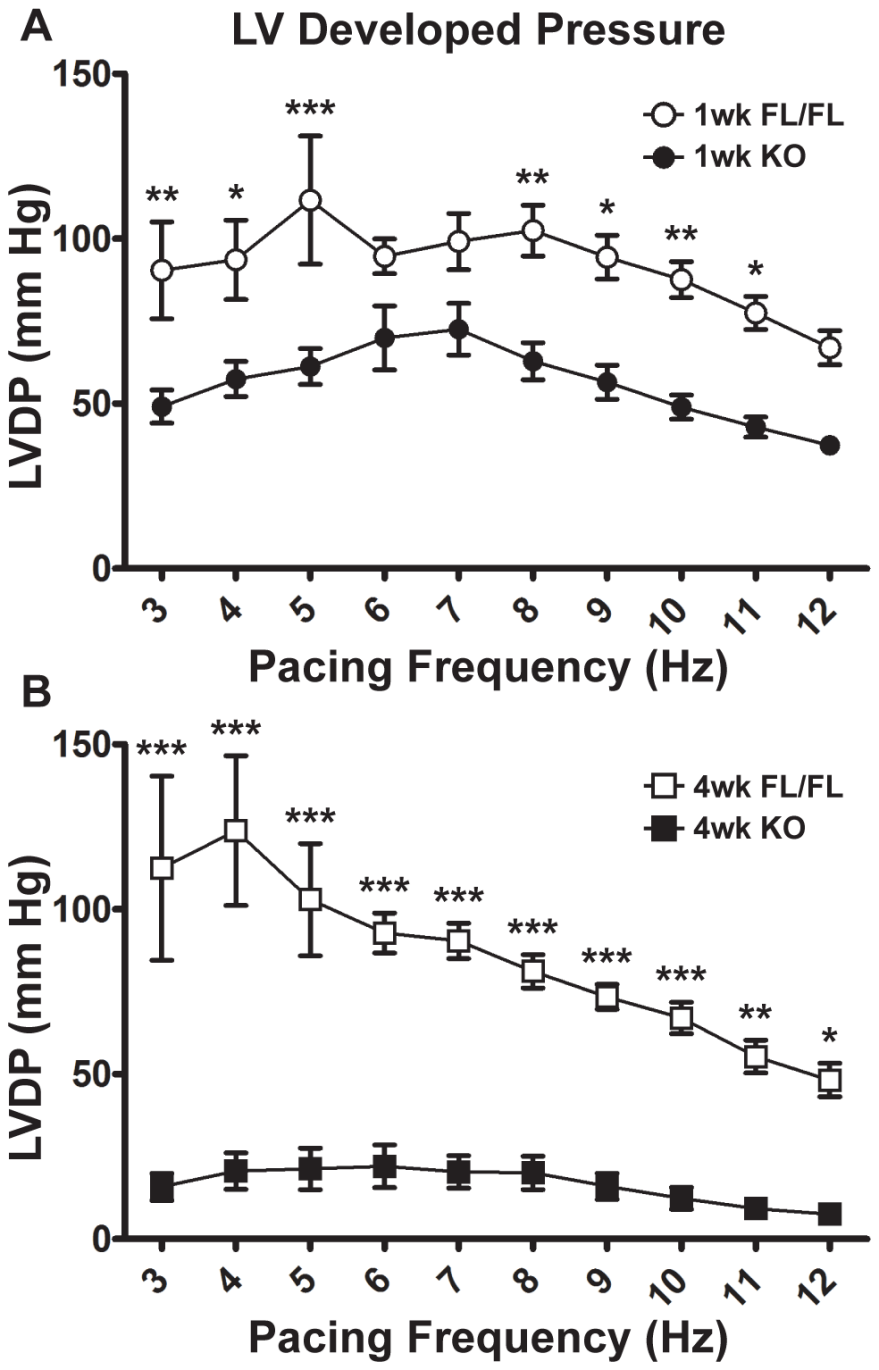
Summary of LV Developed Pressure (LVDP) in isolated Serca2*^fl/fl^* and KO hearts. **A**, 1-week KO vs. 1-week FL LVDP across a wide stimulation frequency range (3–12 Hz). **B**, 4-week KO vs. 4-week FL LVDP. Both 1-week and 4-week FL hearts demonstrated a negative staircase force-frequency response as pacing frequency increased from 7 Hz baseline. 1-week KO vs. 4-week KO: P<0.05 at all pacing frequencies. Some FL hearts could not be paced at very low frequencies: for 1-week FL, the 3 and 4 Hz data points represent N = 3 observations, not N = 4 for all other pacing frequencies. For 4-week FL, 3 Hz represents N = 3 observations, and 4 Hz represents N = 4 observations, rather than N = 5 for all other pacing frequencies. *: P<0.05; **: P<0.01; ***: P<0.001 for FL vs. KO at either 1 week or 4 weeks post-tamoxifen injection, as determined by two-way ANOVA with Bonferroni post-test. Symbols are mean ± SEM.

**Figure 4:**
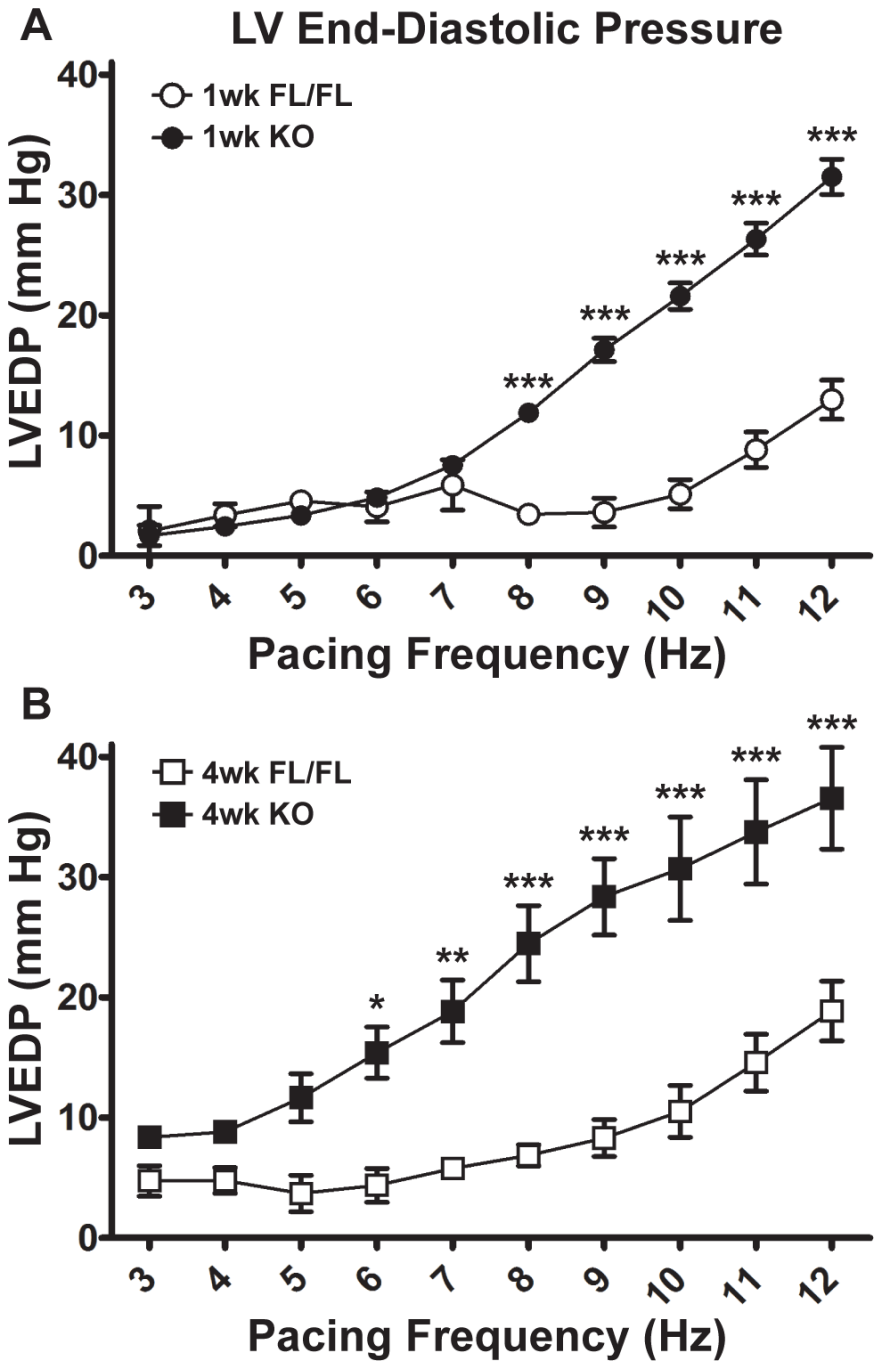
Summary of LV End-Diastolic Pressures (LVEDP) in isolated Serca2*^fl/fl^* and KO hearts. A, LVEDP of 1-week FL and KO hearts from 3–12 Hz stimulation frequency. **B**, LVEDP of 4-week FL and KO hearts from 3–12 Hz stimulation frequency. KO hearts at both 1 and 4 weeks post-tamoxifen injection underwent a pronounced increase in LVEDP as stimulation frequency increased. *: P<0.05; **: P<0.01; ***: P<0.001 for FL vs. KO at either 1 week or 4 weeks post-tamoxifen injection, as determined by two-way ANOVA with Bonferroni post-test.

**Figure 5:**
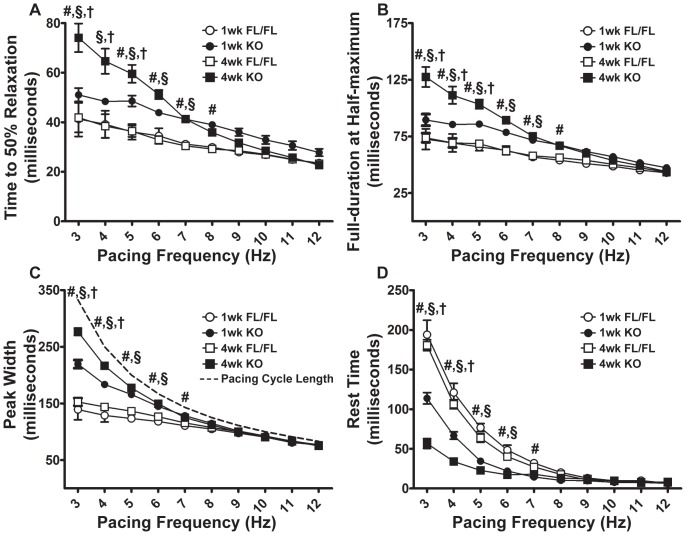
Time to 50% Relaxation (T50R), Full-Duration at Half-Maximum (FDHM), Peak Width, and Rest Time in isolated Serca2*^fl/fl^* and KO hearts. A, T50R; B, FDHM; C, Peak Width; and D, Rest Time in 1-week FL, 1-week KO, 4-week FL, and 4-week KO hearts across 3–12 Hz stimulation frequencies. T50R (A) is the time (in ms) required for pressure to decay from peak to 50% of peak. FDHM (B) is the sum of the time to 50% pressure rise and the time to 50% pressure relaxation and indicates time spent in the contracted state. Peak Width (C) is the time duration between initiation of contraction and return to baseline. C, dashed line indicates the calculated pacing cycle length in milliseconds (PCL = 1000 ms s^−1^ / Pacing Frequency s^−1^) to compare the contractile cycle length to the maximum possible peak width at each pacing frequency. Rest Time (D) is the difference between calculated PCL and Peak Width. Symbols indicate mean ± SEM. #: P<0.05 FL vs KO (1-week). §: P<0.05 FL vs KO (4-week). †: P<0.05 1-week KO vs 4-week KO.

Isolated heart performance at baseline 7 Hz pacing frequency. Values shown, other than time to 50% rise, are also presented with other pacing frequencies in Figures 3, 4, and 5. Values represent mean and SEM of each group's isolated heart performance; values for each heart were determined by averaging 5–10 pressure peaks at each pacing frequency after the heart's performance at that step had stabilized. *: P<0.05; **:P<0.01; ***: P<0.001 for FL vs. KO at either 1 week or 4 weeks post-tamoxifen injection, as determined by unpaired two-tailed *t* test.

Figure 3 summarizes one-week and four-week control and KO heart systolic function (LVDP) across a broad range of pacing frequencies. FL hearts at both time points exhibited a negative staircase force-frequency response as pacing frequency increased above 7 Hz baseline. At one week post-tamoxifen (Figure 3A), KO systolic function was diminished at low and high, but not moderate pacing frequencies (P < 0.05 between KO and FL at 3–5 and 8–11 Hz). At four weeks, however, KO hearts had severe systolic impairment at all pacing frequencies (Figure 3B).

In both one-week and four-week KO hearts, there was a substantial rise in end-diastolic pressure (LVEDP) as pacing frequency increased (Figures 4A and 4B). At four weeks, KO end-diastolic pressures were significantly elevated at lower frequencies compared to one-week KO or FL, but at the maximal pacing frequency of 12 Hz both KO LVEDPs were similar (one-week KO: 31.5±1.5 mm Hg; four-week KO: 36.6±4.2 mm Hg). Further underscoring the relaxation deficit of KO hearts, times to 50% relaxation (T50R) were significantly prolonged in one- and four-week KO hearts relative to FL, particularly at low pacing frequencies (Figure 5A). KO hearts had also a significantly prolonged contracted state (Figure 5B: full-duration at half maximum, FDHM) compared to FL hearts at both time points. Likewise, the peak width (time duration of each pressure peak) progressively increased in one- and four-week KO hearts (Figure 5C). The rest time, defined as the difference between the calculated pacing cycle length and the peak width (PCL = 1000 ms s^−1^/ pacing frequency s^−1^), thus representing the time interval between the end of one peak and the beginning of the next, was elevated in KO hearts at low pacing frequencies (Figure 5D). These measures differed between KO and FL groups at low pacing frequencies and converged as pacing rate increased (7 Hz and above: no significant differences).

**Figure 6:**
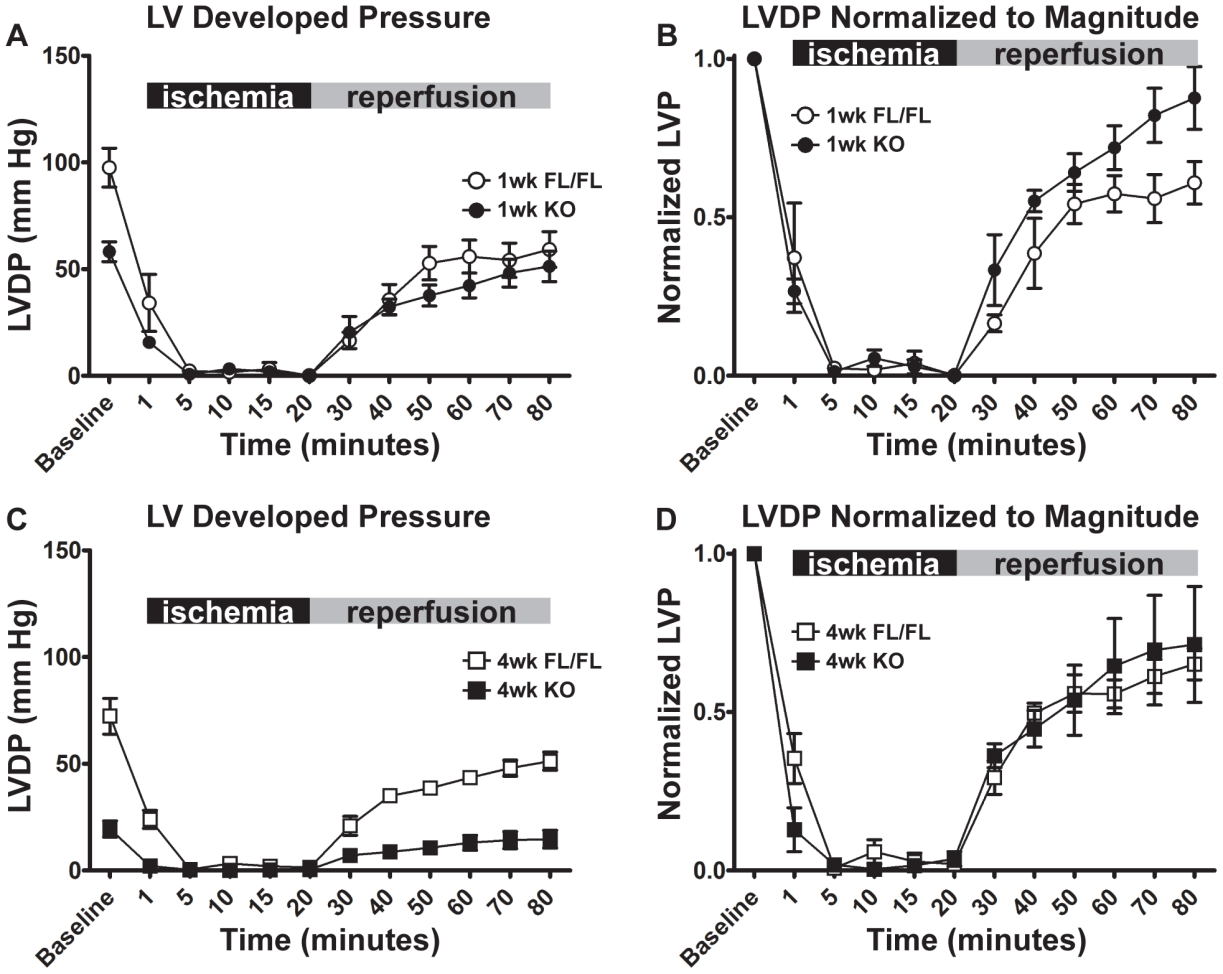
LV Developed Pressures (LVDP) and LVDP normalized to baseline performance during ischemia-reperfusion injury in Serca2*^fl/fl^* and KO hearts. A, LVDP of 1-week FL and 1-week KO hearts at baseline, during ischemia (black bar, minutes 1–20), and during reperfusion (gray bar, minutes 30–80). B, as in (A) except all values normalized to each heart's baseline LVDP. C and D, as with (A) and (B) for 4-week FL and 4-week KO hearts. Baseline values collected immediately prior to the onset of ischemia. One 1-week FL heart encountered a bubble in the perfusion line between pacing and ischemia-reperfusion and became infarcted, so 1-week FL N = 3 for ischemia-reperfusion (Figures 6 and 7). Symbols indicate mean ± SEM. No significant differences between FL and KO at 1 week or 4 weeks post-tamoxifen injection were found by two-way ANOVA.

**Figure 7:**
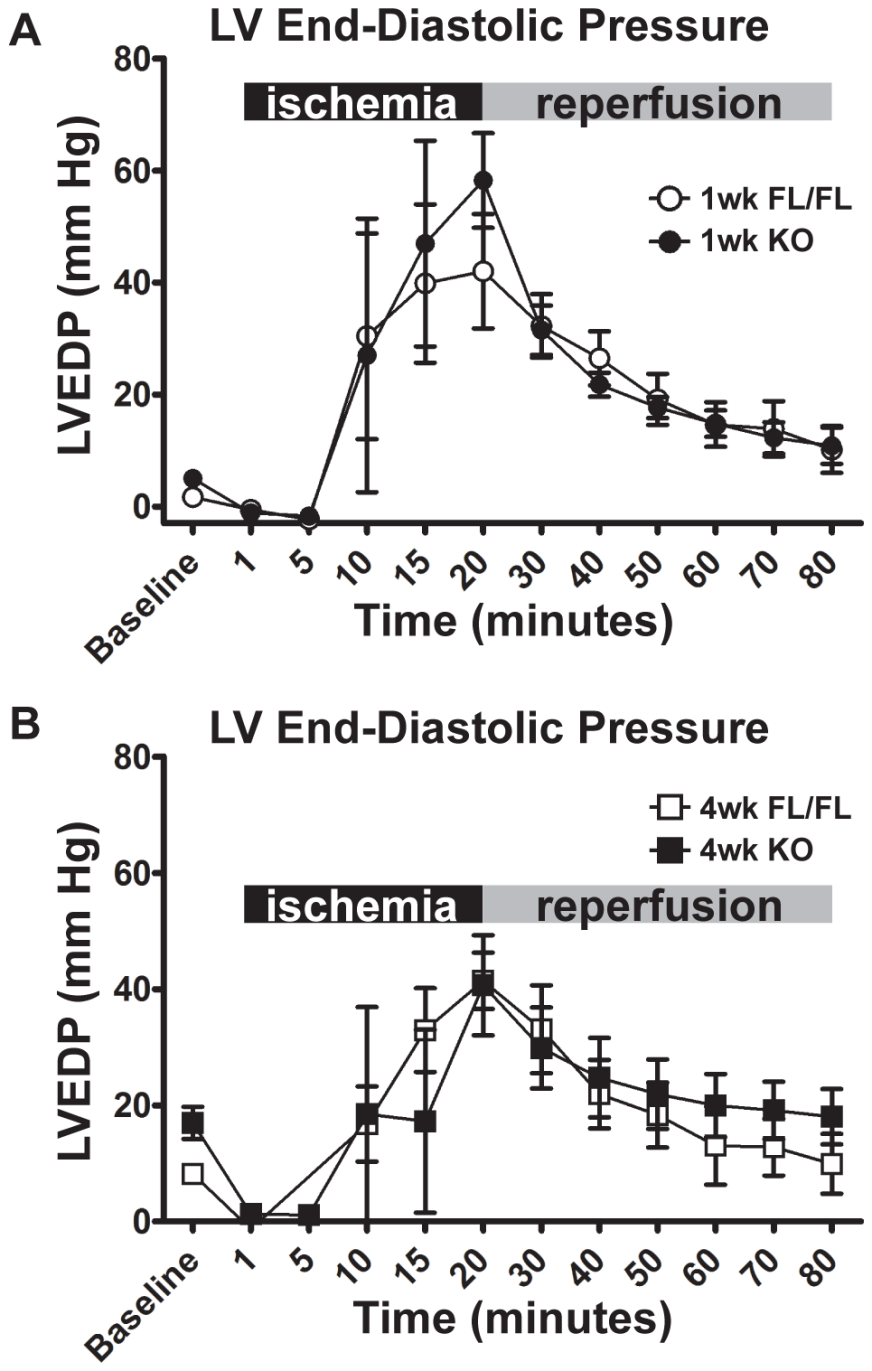
LV End-Diastolic Pressures (LVEDP) during ischemia-reperfusion injury in Serca2*^fl/fl^* and KO hearts. **A**, LVEDP of 1-week FL and KO hearts at baseline, during ischemia (black bars, minutes 1–20), and during reperfusion (gray bar, minutes 30–80). **B**, as (**A**) for 4-week FL and KO hearts. FL and KO groups were not significantly different (P>0.05) at 1 or 4 weeks post-KO, by two-way ANOVA.

Following force-frequency response determination, whole hearts were subjected to ischemia-reperfusion (I/R) challenge to test the hypothesis that the magnitude of I/R-mediated LV pressure dysfunction is modulated by progressive SERCA2a deficiency. At one-week, KO hearts had reduced pre-ischemic baseline LVDP compared to FL hearts, however both groups produced similar absolute developed pressures during reperfusion (Figure 6A). In contrast, at four weeks, KO hearts recovered much lower developed pressures than control hearts during reperfusion. When the developed pressure of each heart was normalized to pre-ischemic baseline values (Figures 6B and 6D), the recovery of KO hearts was not significantly different from FL controls. Also KO and FL hearts developed similar rigor pressure during ischemia, and recovery of LV end-diastolic pressure was not different between groups (Figure 7).

**Figure 8:**
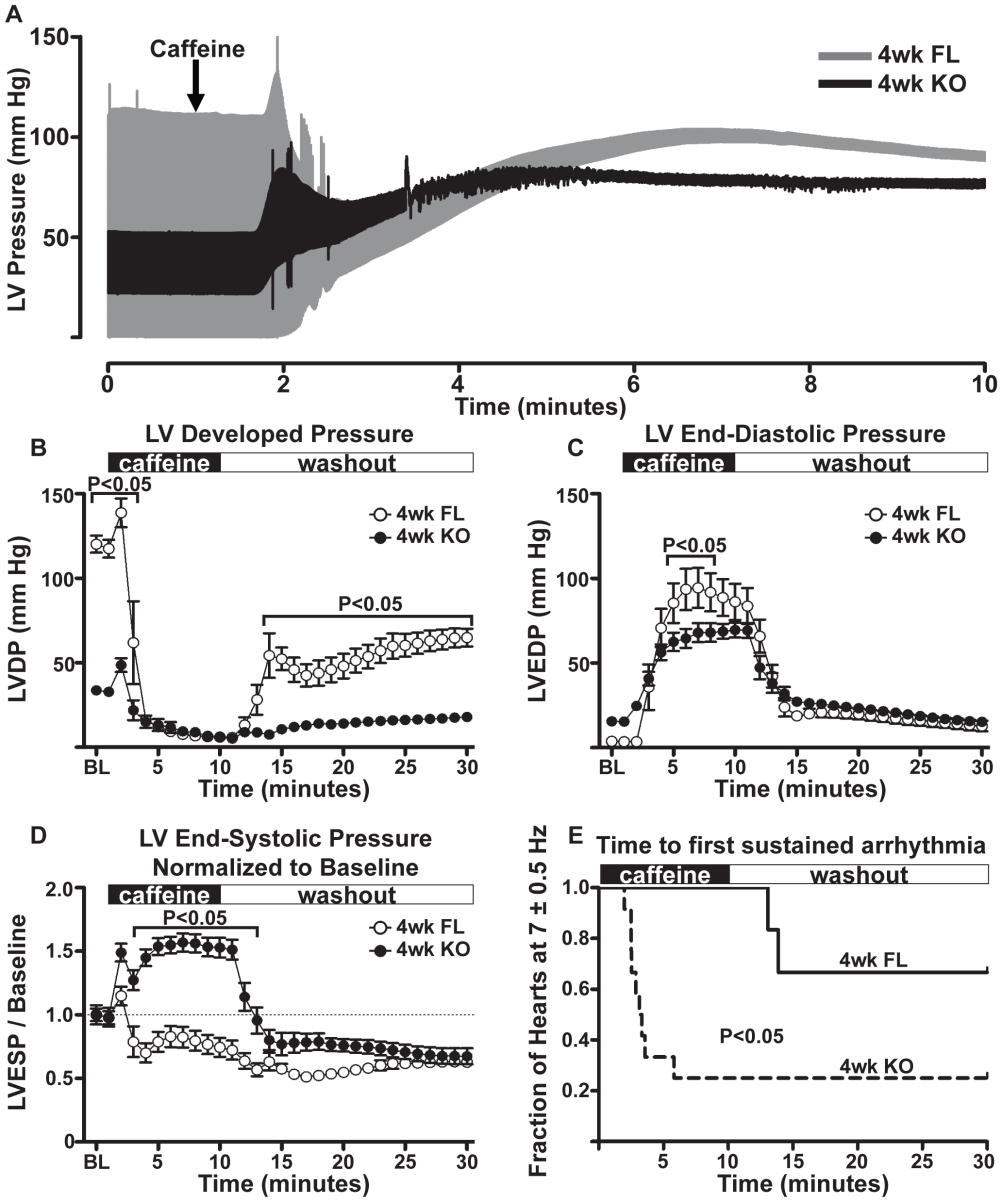
Caffeine perfusion of isolated 4-week FL and KO hearts. **A**, Representative traces of LV pressure from FL and KO hearts spanning the final minute of baseline recording and the first nine minutes of caffeine perfusion. **B**, LVDP of 4-week FL (N = 6) and KO (N = 12) hearts upon perfusion with 10 mM caffeine. Washout with normal KHB began after 10 minutes of caffeine perfusion and proceeded for 20 minutes. Baseline (“BL”) indicates heart performance at the fifth minute of baseline recording, just prior to caffeine perfusion. Dead space in perfusion line took about 1 minute to clear at both switch timepoints. **C**, LVEDP of 4-week FL and KO hearts upon perfusion with 10 mM caffeine. **D**, LV End-Systolic Pressure (LVESP) of 4-week FL and KO hearts upon perfusion with 10 mM caffeine, normalized to baseline level. **E**, Time of survival for 4-week FL and KO hearts from beginning of caffeine perfusion until first 15-second window in which contractile frequency was outside 7±0.5 Hz. Log-rank test: P<0.05 between FL and KO groups. Icons indicate mean, and error bars indicate SEM unless smaller than icon. Panels B, C, and D analyzed by two-way ANOVA. Brackets indicate results of Bonferroni post-tests.

In an additional set of experiments, we sought to further evaluate Ca^2+^ handling in FL and KO hearts by using caffeine to modulate excitation-contraction coupling. Isolated 4-week FL and KO hearts were perfused with 10 mM caffeine, a ryanodine receptor (RyR) activator, to modulate SR Ca^2+^ handling and reveal non-SR dependent contractile function. Caffeine perfusion releases SR Ca^2+^ stores, thus forcing hearts to depend on SR-independent Ca^2+^ handling pathways for contractility. Representative traces of 4-week FL and KO hearts during caffeine perfusion are shown in Figure 8A. Upon perfusion with 10 mM caffeine, isolated hearts initially underwent a brief period of increased contractile force (Figure 8B). Following this initial increase, LV developed pressure diminished in both FL and KO and remained low until removal of caffeine during washout. Upon returning to normal KHB perfusion, FL hearts recovered developed pressure more rapidly than KO hearts. LV end-diastolic pressure increased during caffeine perfusion in both groups, but to a greater extent in FL hearts than KO (Figure 8C). In FL hearts, LV end-systolic pressure (LVESP) decreased during caffeine perfusion, whereas in KO hearts LVESP increased (Figure 8D). During caffeine perfusion, KO hearts were more susceptible than FL hearts to pacing irregularities, defined as a 15-second period in which a heart's contractile frequency deviated outside 7±0.5 Hz. Each heart's time from the start of caffeine perfusion to the first of these events was plotted in Figure 8E. Here, 9 of 12 KO hearts, and 0 of 6 FL hearts, experienced arrhythmias during caffeine perfusion, and 2 of 6 FL hearts experienced arrhythmias shortly after returning to normal KHB perfusion.

## Discussion

This study reports new findings of left ventricular whole heart function after targeted *Serca2* gene disruption with the goal to interrogate organ level performance in the context of progressive and severe SERCA2a protein depletion. Under standard conditions at one week after *Serca2* gene disruption, where cardiac SERCA2a protein content is 32% of control values, isolated whole heart performance is surprisingly near normal. In comparison, under the same standard testing conditions of whole heart function four weeks after *Serca2* gene disruption, where cardiac SERCA2a protein is at near non-detectable levels, there are severe deficiencies in both systolic and diastolic LV function at the level of the isolated intact heart. Cardiac stress testing by increasing pacing rates further and dramatically unmasks relaxation performance deficits at early and late time points after gene disruption and establishes the SERCA2a dose-diastolic performance response relationship. Overall these new findings parallel those obtained previously in isolated myocytes from SERCA2a KO mice[11], [13] and are in contrast to *in vivo* function where heart morphology and whole behavior appear relatively normal. This later point is in keeping with findings of apparent normal echocardiography assessment, at least in the early weeks after severe SERCA2a depletion *in vivo* [11]. Based on whole heart function results reported here it is surprising that SERCA2a KO mice survive well beyond time points where intrinsic whole heart performance is severely compromised.

Our results from the inducible SERCA2a knockout mouse are interesting in the context of previous findings in *Serca2^+/−^* mice, which constitutively express about 60% of normal SERCA2 protein. *Serca2* heterozygous mice have slightly impaired cardiac function, but this does not appear to negatively impact the overall health of these mice[7], [9]. The inducible SERCA2 KO mice[10] offer greater experimental latitude by permitting study of animals at multiple time points between initiation of knockout and the onset of *in vivo* pathology. In this way it is possible to perform detailed study of heart function as SERCA2 protein is specifically and progressively lost from the myocardium. In this context, severe SERCA2a deficiency from 32% of control levels down to near zero levels markedly disrupts heart organ function and yet at ∼ 1,000,000 cardiac pump cycles/day the animal can survive for many weeks before overt cardio-respiratory failure ensues.

The physiological disconnect between the *ex vivo* heart performance and *in vivo* function is evidence that KO mice are capable of compensating for the loss of cardiac SERCA2 protein for up to two months before ultimately failing. Along these lines, recent reports on isolated rabbit hearts exposed to the SERCA2 inhibitor, thapsigargin, also show a surprising extended time of whole heart function that too appears to uncouple isolated myocyte SR Ca^2+^ flux and whole-heart contractility[16].

As of yet the mechanism allowing for this compensation is not clear, although there are several changes shown to occur following *Serca2* disruption that may support heart function in the absence of robust SR Ca^2+^ flux. For example, there are modest increases in the expression and activity of the L-type Ca^2+^ channel, the Na^+^-Ca^+^ exchanger, and the plasma membrane Ca^2+^ ATPase[13]. In addition, Na-K ATPase expression and activity is decreased[13], and serum norepinephrine is elevated in KO mice[11]. Collectively, these changes prompt the hypothesis that *Serca2* KO cardiac performance *in vivo* is maintained by enhancing trans-sarcolemmal Ca^2+^ transport while SR Ca^2+^ handling is reduced. KO myocytes exhibit greater Ca^2+^ transients than FL when stimulated during caffeine perfusion[18]. The contractile performance of isolated KO hearts perfused with caffeine, however, suggests that trans-sarcolemmal Ca^2+^ flux is unlikely to account for preserved systolic function after *Serca2* disruption. Systolic performance in FL and KO hearts is nearly identical during caffeine perfusion. End-diastolic pressures are lower in KO hearts during caffeine perfusion, suggesting that increased NCX and PMCA levels found 4 weeks post-KO can help sustain diastolic performance. KO hearts tend to deviate from the programmed stimulation frequency, 7 Hz, soon after the application of caffeine, indicating that this increased dependence on trans-sarcolemmal Ca^2+^ fluxes can sensitize KO hearts to arrhythmic behavior[19]. This finding is notable because <1 week KO mice exhibit fewer ventricular extrasystoles than FL mice in telemetry studies[14]. This difference is likely accounted for by the progressive nature of this Serca2 KO model, as the 4-week KO hearts used in this study have diminished SR function and greater NCX activity[13] than <1 week KO hearts[14]. Therefore, compensatory changes in Ca^2+^ handling mechanisms of *Serca2* KO hearts may contribute to comparatively preserved end-diastolic pressures, but not systolic performance, during caffeine exposure. Increased dependence on Ca^2+^ current, however, may render KO hearts susceptible to arrhythmias. Additional studies will be required to further specify how KO hearts maintain systolic performance *in vivo* as SR function is gradually abolished.

Loss of SERCA2 expression and activity is well documented within a wide variety of cardiac pathologies in human patients. However, in this context it is interesting to note that patients with Darier's disease, which owing to *Serca2* loss-of-function mutations are functional Serca2^+/−^ heterozygotes, show no sign of cardiac disease or dysfunction[20]. This observation supports the apparent disconnection between in vivo cardiac function, cardiac SERCA2 content/activity, and isolated organ and cell function.

Another motivation for studying *Serca2* KO mice was to assess the relationship between SR function and ischemia-reperfusion injury. Earlier reports of altered SR function using phospholamban deficient mice reported much more severe I/R-mediated heart performance deficits than in controls[17]. We speculated that a converse model whereby SR Ca^2+^ uptake and load are reduced would have opposite effects and paradoxically could be beneficial in terms of relative LV functional deficits in I/R. In 1-week KO hearts with SERCA2a reduced to 32% of control, mean values for LVDP tended to be higher than controls but this did not reach statistical significance by ANOVA analysis. Thus, although increasing SR Ca^2+^ load by phospholamban ablation severely impairs recovery from ischemic injury[17], decreasing SERCA2a content to 32% or less appears not to confer any benefit to recovery.

In conclusion, these findings show that progressive loss of SERCA2 protein from heart following inducible knockout initiates rapid, severe decline in the contractile performance of isolated whole hearts. Although the mechanisms allowing for sustained *in vivo* cardiac function and survival are currently unclear, identifying the manner in which hearts compensate for severely diminished SR Ca^2+^ flux could prove highly beneficial to our understanding of the interrelationship between SR Ca^2+^ derangements and cardiac disease.
